# Evaluating teen driving knowledge and behaviors following educational outreach

**DOI:** 10.1186/s40621-020-00255-0

**Published:** 2020-06-12

**Authors:** Kathy Monroe, Michele Nichols, Marie Crew, Leslie Brown, William King

**Affiliations:** 1 Department of Pediatrics, Division of Pediatric Emergency Medicine, University of Alabama Birmingham, Birmingham, AL USA; 2 SafeKids of Alabama, Children’s of Alabama, 1600 7th Ave So, Suite 110 CPP, Birmingham, AL Al 35233 USA

**Keywords:** Teen driving, Education, Risky behaviors

## Abstract

**Background:**

Teen driving educational events are an effective strategy to increase adolescent drivers’ awareness of safe driving practices. The objectives of this study were to evaluate changing rates of self-reported driving practices and knowledge of the state Graduated Driver Licensing laws (GDL) by teens over a nine-year period in a single state.

**Methods:**

This was a prospective observational study of high school students ages 14 to 19 years old. Paper surveys were sent to the high schools participating in teen driving educational events (9 schools in 2009 and 4 schools in 2018). Students in those schools completed surveys prior to the events. Students completing the anonymous survey were invited to the event. Questions evaluated awareness of state GDL and safe and risky driving behaviors. Statistical comparisons of survey answers from 2009 to 2018 were analyzed using the z test of proportions (2 tailed, alpha 0.05).

**Results:**

A total of 397 students participated in 2018 with ages ranging from 14 to 19 years. Racial distribution was 81% white, 14% black, and there were 57% female participants. Only 69% (*n* = 273) reported “always” wearing their seatbelt. When asked about high risk behaviors, 78% (*n* = 309) of adolescents reported they personally “never” text while driving; 97% (385); never drive after drinking, and 87% (*n* = 344) never ride with someone who has been drinking. Compared to 2009 participants (1304 students, 9 schools from central part of state), the students in 2018 (4 schools scattered across state) reported wearing seatbelts “always” (*n* = 69% vs 39%; *p* < 0.001); “never texting while driving” (78% vs 33%; p < 0.001); and “never drinking and driving” (97% vs 88%; *p* < 0.001). No significant difference in rates of students having taken a driving education class nor driving over speed limit were reported.

**Conclusion:**

Results are encouraging that participants in 2018 report more use of seatbelts, less texting while driving, less drinking while driving and lower numbers of being in MVC than in 2009. However, rates of high-risk driving behaviors are still concerning.

## Background

Motor vehicle crashes (MVCs) are the number one cause of death for teens in the U.S. (Center for Disease Control and Prevention n.d.). The risk of motor vehicle crashes is higher among 16- to 19-year-olds than among any other age group. In fact, per mile driven, teen drivers 16 to 19-years old are three times more likely than drivers 20 years and older to be involved in a fatal crash. (Insurance Institute for Highway Safety (IIHS) 2010). Alabama, the state in which this study occurred, consistently ranks among the worst 10 states in the nation for teen driving fatalities (Teen driving safety 2019). Graduated Driver Licensing (GDL) laws were enacted in Alabama in 2002, with strengthening of those laws in 2010 and again in 2015. According to the state Department of Public Health, alcohol, non-seatbelt use, and distractions are the three primary contributing factors to teen driving deaths (Alabama Department of Public Health n.d.).

Inexperience and immaturity both contribute to high crash rates involving teen drivers (McKnight and McKnight 2000). Adolescent drivers tend to engage in numerous risky behaviors such as driving over the speed limit, which has been found to significantly correlate with a greater risk for crashes (Elander et al. 1993). They are more likely to engage in other risk-taking behaviors as well. According to 2004 National Highway Traffic Safety Administration (NHTSA) data, 17% of young drivers 16–20 years old had a blood alcohol concentration at or above 0.08%, the level at which all states define drunk driving (National Highway Traffic Safety Administration 2005). According to various NHTSA sponsored state and national safety belt surveys, youth 16–24 years old are observed wearing safety belts at rates 5–15% below rates for those older than 24 years (Williams et al. 2003; Glassbrenner 2003). Numerous surveys conducted in high school parking lots indicate typical teen belt use is about 50–60%, depending upon the state and the school (McCartt and Shabanova 2002), but that rates of seat belt use among teens vary dramatically based on age, gender, race, and urban/rural setting and region of the country.

These findings suggest that not only are interventions to increase seat belt use by young people greatly needed, but also that targeted messages among teens—based on age, gender, race, and urban/rural and regional differences (Juarez et al. 2006). There has been acknowledgement that strategies targeting the general population do not necessarily have the same effect on all segments of the population (Juarez et al. 2006). Graduated Driver Licensing laws, driving instructional classes, parent teen driving contracts, and pediatrician counseling have all been suggested as means of influencing teen driving fatalities. We began hosting crash course teen driving events at high schools in our state in 2009 and have continued these events to present day. Since our state has high teen driving fatality rates, our objective in this study was to determine the prevalence of high risk driving behaviors (including non-use of seat belts, texting and drinking while driving) among area teens in 2018 and to evaluate changing rates of self-reported risky driving practices and knowledge of the state GDL by teens compared to those in 2009.

## Methods

This was a prospective observational study of high school students ages 14 to 19 years old. We surveyed the students to assess knowledge of the Alabama GDL law and prevalence of high-risk driving behaviors in 2018 and compared those to the original responses from 2009. This study utilized a teen driver questionnaire to provide estimates and baseline information on teen driving behaviors in our state (Table 1). The questionnaire was adapted from the Centers for Disease Control and Prevention (CDC’s) National Youth Risk Behavioral Survey (NYRBS) (Eaton et al. 2012). Questions regarding behavior as drivers and passengers in the car were included verbatim. In addition, questions were included to determine if the students had discussed safe driving with either parents or with their primary care physician. The added questions were written in similar style to the NYRBS questions and were pilot tested with a small group of adolescents for readability. The completion of a driver education course was also documented by self-report from the participants. Our survey also included questions about the Alabama GDL law including. Institutional Review Board approval was obtained.

**Table 1:** Teen Driving Survey Questions

Survey Questions	
1. How old are you? A. 14 years old or younger D. 17 years old B. 15 years old E. 18 years old or older C. 16 years old	
2. What is your sex? A. FemaleB. Male	
3. What is your race? (**Select one or more responses.)** A. American Indian or Alaska NativeD. Native Hawaiian or Other Pacific Islander B. Asian E. White C. Black or African American F. Hispanic or Latino	
4. Has your doctor talked to you and/or your parents about: (select all that apply) ___ wearing seatbelts___ dangers of drinking and driving ___ dangers of texting while driving ___ dangers of drug use with driving ___ speeding___ number of passengers that should ride in car with you ___ driving contract ___ haven’t seen my doctor in the past 3 yrs. ___ none of these	
5. How often do you wear a seat belt when **riding** in a car? A. Never B. Rarely C. Sometimes D. Most of the time E. Always	
6. What are reasons why you don’t always wear your seatbelt? __ don’t think they work__ afraid if I have an accident I’ll be stuck __ don’t have one__ forget to put it on ___ just don’t want to__ it messes up my clothes __ none of my friends do__ I always wear one	
7. How often in the past 30 days, have you observed your parents text while driving? A. Never B. Rarely C. Sometimes D. Most of the time E. Always	
8. How often do your parents wear a seatbelt? A. Never B. Rarely C. Sometimes D. Most of the time E. Always	
9. During the past 30 days, how many times did you **ride** in a car or other vehicle **driven by someone who had been drinking alcohol?** A. 0 times B. 1 time C. 2 or 3 times D. 4 or 5 times E. 6 or more times	
10. During the past 30 days, how many times did you **drive** a car or other vehicle **when you had been drinking alcohol?** A. 0 times B. 1 time C. 2 or 3 times D. 4 or 5 times E. 6 or more times F. I do not drive/have a permit or license	
11. During the past 30 days how many times did you **ride** in a care or other vehicle **driven by someone who was texting while driving?** A. 0 times B. 1 time C. 2 or 3 times D. 4 or 5 times E. 6 or more times	
12. During the past 30 days how many times did you **drive** a car or other vehicle while **texting**? A. 0 times B. 1 time C. 2 or 3 times D. 4 or 5 times E 6 or more times F. I do not drive/have a permit or license	
13. How long after you obtained your license were you allowed to drive with friends in the car? A. ImmediatelyB. DaysC. WeeksD. MonthsE. Years F. I do not drive/have a permit or license	
14. How much time have you spent discussing the dangers of driving with your parents? A. noneB. < 1 h C.1–3 h D. 4–6 h E. > 6 h	
15. Have you ever taken a driving class? YesNo If yes, was it a class at your school? Was it a class at another site?	
16. Have you ever been involved in a car crash as a driver? A. Yes b. No c. Do not drive	
17. Which of the following have you discussed with your parents?(select all that apply) __ wearing seatbelts __dangers of drinking and driving ___ dangers of texting while driving ___dangers of drug use while driving ___speeding____number of passengers that teen can have ___rules when friends are in car____ driving contract ___ none of these	
18. Do you routinely drive 5–10 miles over the speed limit? A.Yes B. No C. Do not drive	
19. In the past thirty days, have you driven while under the influence of drugs? A. YesB. NoC. Do not drive	
20. Are you aware of the graduated driver’s license laws?Yes No	
21. Do you ever use your cellphone (for any reason) while driving? A. YesB. NoC. Don’t have a cell phoneD. Don’t drive yet	
22. If yes, what could influence you to give up your cell phone while driving (or what might influence you when you begin driving to not use cell phone)? (check all that apply) ___ if your license could be taken away if caught ____ if friends gave up their phone ___ if there was a law against using phone ____ could get insurance discount ___if parents knew every time you used phone ____ nothing could convince me to stop using my cell phone ___ don’t use cell phone while driving	
23. Have you ever signed a driver safety contract? A. YesB. NoC. Never heard of one If no, would you be interested in one	
24. If you are aware of the teen driving contracts but haven’t signed one, why not? A. I don’t drive yet B. No one has ever asked me to sign a contract C. I refused	
25. If you have signed a driver safety contract, did you sign contract: a. After my parents found out I was speeding b. After I had a car crash c. After my parents found out I had driven after drinking d. After my parents found out I had driven while texting e. Before I started driving f. None of the above g. I have not signed a driving contract	
26. According to Alabama Law, what is the curfew hour for teen drivers under the graduated drivers license rules: a. 8 pm b. 9 pm c. 10 pm d. 11 pm 3. 12 am

**Table 2:** Comparison of Participant Responses from 2018 compared to Responses in 2009

Question	2009	2018	Z score	***p***-value
N=	1304	397		
In the past 30 days have you “Always” worn a seatbelt when driving?	504 (39%)	273 (69%)	**8.3**	**< 0.001**
In the past 30 days how often have you text while driving (NEVER)	441 (33%)	309 (78%)	**8.9**	**< 0.001**
In the past 30 days how often have you driven after drinking alcohol? (NEVER)	1146 (88%)	385 (97%)	**4.5**	**< 0.001**
Have you ever taken a driving class	765 (59%)	249 (63%)	**−1.5**	**0.14**
In the past 30 days have you routinely driven 5–10 miles over the speed limit?	764 (59%)	218 (55%)	**−0.12**	**0.9**

We began hosting crash course teen driving events at 9 high schools in the central part of Alabama 2009. With the 2009 driving event, paper surveys were distributed prior to the teen driving educational event to evaluate self-reported driving behaviors and knowledge of GDL from the high school students. The educational event following the surveys was a 1 day event in which a variety of speakers addressed the students including a state trooper, a mother of a child who died in a motor vehicle crash, and a young adult who survived a motor vehicle crash, but is paralyzed from the crash. There were interactive sessions including driver’s simulation, drunk goggle demonstrations, and a jeopardy-style game about GDL awareness.

For this current study, paper surveys were sent to four high schools participating in the teen driving educational events scattered throughout Alabama. Inclusion criteria were students ages 14 years and older in one of the participating schools. A letter was sent to students’ parents informing them of the survey and allowing them to have their child opt out of participation if they so desired. The surveys were then sent to the participating schools and the questionnaire was distributed to high school students for completion prior to the educational event. Students completing the survey were invited to the event, but since the survey was anonymous, it is possible some students completed survey, but did not attend event. Participation in the survey was voluntary. Consent for the survey was by completion and return of survey (opt out method). Consent for event was written consent by parents to the school to allow them to travel to the event. Respondent identities were concealed as all surveys were anonymous. There was no individual incentive to participate. In 2009 there was a $500 award to the school with the most surveys.

In 2009 there were nine schools from the central portion of the state participating in one event. In 2018 there were four schools from across the state (none in the central portion that participated in 2009) participating in four separate events. We calculated descriptive statistics and compared survey answers from 2009 to 2018 using the z test of proportions using a 2 tailed alpha level of 0.05 as significant. All analyses were conducted in Epi Info 7.0.9.7 (2/9/2012, CDC, Atlanta Georgia, 2011).

## Results

A total of 397 students from four schools across Alabama participated in 2018. Ages of students ranged from 14 years (*n* = 44);15 years (*n* = 171); 16 years (*n* = 101); 17 years (*n* = 67), and 18 or older (*n* = 13), with 57% female (*n* = 228). Racial distribution was white (*n* = 323; 81%); black (*n* = 56; 14%); and Hispanic (*n* = 12; 3%). Only 69% (*n* = 273) reported “always” wearing their seatbelt, and when asked if any physician had ever talked to the adolescent about wearing seat belts only 168 (42%) said yes. Only 63% (*n* = 249) reported having taken a driving class in the past. A total of 55% (*n* = 218) reported routinely going more than 10 miles above speed limit.

When asked about high risk behaviors, 78% (*n* = 309) of adolescents reported they personally “never” text while driving, 97% (*n* = 385) participants stated they never drive after drinking, and 87% (*n* = 344) stated they never “ride with someone whose been drinking.” Students (*n* = 225), reported seeing their parents wear seatbelts “always” 64% of time. When asked if their parents text while driving, only 26% said “never” and 15% said “most or all the time.” When asked if their parents had ever discussed safe driving with them, 15% reported no time was spent; 41% < 1 h, 27% 1–3 h, 8% said 4–6 h, and 9% over 6 h. A total of 230 (58%) were aware of the GDL laws, with only 26% correctly answering the curfew question.

In 2009, there was one teen driving educational event with nine schools participating from one county in central portion of the state. There were 1304 respondents to the survey in 2009. These schools did not overlap with the four schools participating in 2018. Comparing to 2009 participants, the students in 2018 reported wearing seatbelt “always” (*n* = 69% vs 39%; *p* < 0.001); “never texting while driving” (78% vs 33%; p < 0.001); and “never drinking and driving” (97% vs 88%; *p* < 0.001) (see Table 2). No significant difference in rates of having taken a driving class nor driving over speed limit was demonstrated between the 2 years.

## Discussion

This study gives insight into current teen driving behaviors in one state, and how those compare to self-reported teen driving behaviors from 10 years ago. The students in 2018 reported higher rates of “always wearing a seatbelt,” “never texting while driving,” and “never drinking and driving” compared to the students in 2009. Motor vehicle crashes remain the leading cause of teen deaths despite preventive efforts (Missikpode et al. 2018). The need for innovative behavioral interventions for teen drivers has been made clear, and previous studies have shown benefit from school based programs (Unni et al. n.d.; Hafner et al. 2019). Local surveillance is important in guiding interventions and the NYRBSS is an important tool in gaining insight into teen drivers’ beliefs and behaviors. Our events combined surveillance of behaviors with school based educational outreach.

Results from our surveillance study were encouraging in that participants in 2018 report more use of seatbelts, yet only 69% report always using seatbelts, so more work needed. Of the teens (13–20 years old) who died in passenger vehicle crashes in 2013 approximately 56% were not wearing a seat belt at the time of the crash (National Highway Traffic Safety Administration n.d.). Research shows seat belts reduce serious crash-related injuries and deaths by about half, (Kahane 2013) yet compared with other age groups, teens have the lowest rate of seat belt use. In 2013, only 55% of high school students reported they always wear seat belts when riding with someone else (Centers for Disease Control and Prevention 2013). Our state has had a primary seatbelt law since 1991, yet we continue to see many teens who do not routinely wear their seat belt with 31% of our survey respondents reporting inconsistent seat belt usage. The fine for not wearing a seatbelt in our state is only twenty-five dollars.

Cell phones have received much attention as a source of driver distraction. Our state has had a texting ban in effect since 2012, and the Graduated Driver Licensing law specifically bans any handheld device for teen drivers (Beck et al. 2002). Despite this legislation, 31% of survey respondents reported texting while driving in the past month in 2018 survey. There was a significant improvement; however, with 78% reporting never texting while driving in 2018 compared to 2009 when only 33% said they never text while driving.

At all levels of blood alcohol concentration (BAC), the risk of involvement in a motor vehicle crash is greater for teens than for older drivers (Ginsburg et al. 2009). In a national survey conducted in 2013, 22% of teens reported that, within the previous month, they had ridden with a driver who had been drinking alcohol (Centers for Disease Control and Prevention n.d.). Among students who drove, 10% reported having driven after drinking alcohol within the same one-month period (Centers for Disease Control and Prevention n.d.). In our study, the reported rates of “never” drinking and driving had improved from 88 to 97% of respondents in 2009 to 2018 respectively. Unfortunately, we found no change in those reporting routinely driving over the speed limit, with over 50% of respondents reporting routinely driving more than 10 miles over the speed limit in both groups.

The results of our study reveal an alarming number of risky behaviors in teen drivers. The National Young Risk Behavior Survey questioned 9th, 10th and 11th graders (14,15 and 16 years old). While our participants were primarily 15 and 16 years old, we also surveyed 17 and 18-year olds. Although not a direct comparison due to the slight age differences, our numbers were comparable to the 2011 NYRBS. Nationwide, 7.7% of students rarely or never wear a seatbelt, 8.2% drove in the last 3 days after consuming alcohol, and 32.8% texted while driving in the last 30 days (McKnight and McKnight 2000). Our local respondents reported 18, 1, and 31% respectively. It is clear our teen drivers are participating in risky driving behaviors at an alarming rate, even when compared to national data.

Despite the presence of a GDL law in our state since 2002 with revisions in 2010 and 2015, our survey indicates many teens do not follow the guidelines of this law. Besides the GDL, our state has had a primary seat belt law since 1997, yet only 69% of respondents said they always wear their seatbelt when driving. In addition, 55% of respondents reported they routinely drive 5–10 mph over the speed limit. It is apparent that legislation is important, but there are additional factors involved in teen driving safety.

### Limitations

One limitation of any self-reported survey is the risk of responder bias. As a result, participants may not answer truthfully, but this survey was anonymous and voluntary, so participants had no reason to falsify answers. There may also be bias in the type of student who would complete this kind of survey. We also did not specifically ask if the student respondent was a driver, thus we were not able to further analyze the responses by driver status. There is also the potential for recall bias as this survey asks respondents to remember the frequency of certain behaviors and actions. However, the survey questions were either taken directly from the NYRBS or created in similar style, and the NYRBS tool has been validated for reliability (Brenner et al. 1995). The environment for some risk driving behaviors (e.g. texting) are different in 2018 compared to 2009, so these results should be interpreted with some caution. This study was conducted in one southern state with a significant teen driving fatality rate; therefore, the results may not be generalizable to teenagers in other states. Also, the surveys were not distributed to all Alabama teenagers, and only represents the subset of students attending one of the high schools participating in the educational events. The schools who participated in 2018 were not the exact same schools that participated in 2009; however, the same survey for the same age population in the same state was utilized. Thus, we can only describe general differences in responses between the 2 years, but not specific changes between the same student or among the same schools.

## Conclusions and future directions

Teen driving death and injury is a significant problem in our nation. Results are encouraging that participants in 2018 report more use of seatbelts, less texting while driving and less drinking while driving than in 2009. However, rates of seatbelt use are still only 69%, and low rates of physicians discussing seatbelts were still reported. Also, an alarming number of teens still report risky driving behaviors in this study. Some state-based interventions such as the graduated driver licensing law, safe driving media campaigns, and a Teen Driving Toolkit for state pediatricians have already been instituted in Alabama, and the teen driving educational events have expanded to cover the different areas of the state. Persistent efforts to increase public awareness of teen driving safety issues is indicated.

## Data Availability

The datasets used and/or analyzed during the current study are available from the corresponding author on reasonable request.

## Abbreviations

BAC: Blood alcohol concentration
CDC: Centers for Disease Control and Prevention
GDL: Graduated driver’s license
MVC: Motor vehicle crash
NHTSA: National Highway Transportation Safety Administration
NYRBS: National youth risk behavior survey
U.S.: United States
